# Characterization of National Immunization Programs in the Context of Public Health Emergencies: A Case Study of 13 Countries in the WHO Africa Region

**DOI:** 10.3389/fpubh.2021.736532

**Published:** 2021-09-28

**Authors:** Viola Chepkurui, Edina Amponsah-Dacosta, Eposi Christiana Haddison, Benjamin Mugo Kagina

**Affiliations:** ^1^Faculty of Health Science, School of Public Health and Family Medicine, University of Cape Town, Cape Town, South Africa; ^2^Vaccines for Africa Initiative, School of Public Health and Family Medicine, University of Cape Town, Cape Town, South Africa; ^3^Saa Health District, Centre Regional Delegation of Public Health, Saa, Cameroon

**Keywords:** public health emergencies, national immunization programs, decade of vaccines, WHO Africa Region, African countries

## Abstract

Multiple public health emergencies (PHEs) experienced annually in the World Health Organisation (WHO) Africa region affect the provision of health services, including immunization. However, there is limited information on the performance of national immunization programs (NIPs) in WHO Africa countries that experience PHEs. This study assessed PHEs (armed conflicts, disasters, and disease outbreaks) and the performance of NIPs using global and regional immunization targets outlined for the Decade of Vaccines. Thirteen beneficiary countries of PHE mitigation funds from the African Public Health Emergency Fund were used as case studies. Data on PHEs and immunization indicators between 2010 and 2019 in selected countries were extracted from different PHE databases and the WHO/UNICEF immunization database, respectively. The data were stratified by country and summarized using descriptive statistics. Mann-Whitney *U* test was done to determine the association between the frequency of PHEs and the performance of NIPs. There were 175 disease outbreaks, 288 armed conflicts, and 318 disasters in the examined countries between 2010 and 2019. The Democratic Republic of Congo had the highest total PHE count (*n* = 208), while Liberia had the lowest (*n* = 20). Only three of the 13 countries had a median coverage value for the third dose of the combined Diphtheria, Tetanus, and Pertussis vaccine (DTP3) that had attained the target for ≥90% immunization coverage. Higher counts of armed conflict and total PHEs were associated with not meeting immunization targets for national DTP3 coverage of ≥90% and Maternal and Neonatal Tetanus elimination, *p* < 0.01. It was clear that in the WHO Africa region, PHEs are prevalent, irrespective of a country’s level of immunization maturity, and have the potential to derail the progress of NIPs in the absence of effective interventions. As we transition toward the Immunization Agenda 2030, we recommend that the WHO Africa region prioritizes interventions to mitigate the impacts of PHEs on NIPs.

## Introduction

Public Health Emergencies (PHEs) are recognized as a major threat to global public health security (1). Many countries experience different types of PHEs including infectious disease outbreaks, natural disasters^1^ (e.g., floods), and human-induced hazards (e.g., armed conflicts) (2). In the WHO Africa region, more than 100 PHEs are reported annually (3). A PHE is described as a situation that threatens the health, safety, and well-being of a large number of people and which often necessitates substantial multi-sectoral assistance (2, 4). For instance, the recent unprecedented occurrence of the Coronavirus Disease (COVID-19) pandemic, which is classified as a PHE of international concern (5), has highlighted more than ever that PHEs do transcend international boundaries and have negative impacts on various facets of society including public health (2, 3). The impact PHEs have on public health cannot be understated, given they cause widespread disruption to both the broader health system and provision of health services (6–8) including immunization (9, 10).

The disruption to national immunization programs (NIPs) experienced in countries with PHEs has been associated with suboptimal immunization coverage rates (6, 11, 12). The plummeting coverage witnessed during PHEs is facilitated by reduced access to immunization services, destroyed and disrupted vaccine logistic systems, depleted and diverted financial and human resources for immunization, and reduced trust toward immunization (6, 11, 12). Many of these factors have eminently featured during the COVID-19 pandemic (13–15). The sub-optimal immunization coverage rates, also, create large pockets of unimmunized and under-immunized children and adults thereby lowering herd immunity (16, 17). The compromised herd immunity acts together with other PHE co-existing factors like poor sanitation and overcrowding to provide ideal conditions for outbreaks of Vaccine-Preventable Diseases (VPDs) (18–20). Due to such frailties, countries affected by PHEs are reported to lag in meeting global and regional immunization targets aimed at control, elimination, and eradication of VPDs (21, 22).

The Global Vaccine Action Plan (GVAP) was developed in 2011 to actualize the vision of the Decade of Vaccines (DoV) for having a world where individuals and communities will be able to enjoy lives free from VPDs by 2020 (23). The Regional Strategic Plan for Immunization (RSPI) is a WHO Africa region contextualized immunization framework adopted from the GVAP with goals for attainment by 2020 (24). Although through the two immunization frameworks the WHO Africa region has achieved substantive gain in immunization e.g., the successful eradication of wild poliovirus disease (25), many targets remain unmet, owing to among other factors the prevalence of PHEs (26). During the DoV there has been growing concern on the possible role PHEs play in delaying achievement for set immunization targets (22, 26). According to reports by the WHO, Strategic Advisory Groups of Experts in immunization (SAGE), PHEs are recognized as one of the major shifts during the decade and a key driver of inequity in immunization access between and within countries in the region (22). With the end in sight of the DoV in 2020, there is need to take stock of immunization performance in countries experiencing PHEs. The evidence generated would advance the understanding that is critical in strengthening NIPs in the context of PHEs.

In as much PHEs have been recognized to have repercussions on NIPs, inadequate focus has been given on synthesizing evidence in PHE-affected settings in the WHO Africa region. Few studies have attempted to describe immunization in countries affected by PHEs, however, a majority are restricted to single types of PHEs, especially armed conflict and single immunization indicators (6, 11, 27). To the best of our knowledge, this will be the first study that aims to characterize NIPs in the WHO Africa region experiencing PHEs, using three different types of PHEs (i.e., armed conflicts, disease outbreaks, and disasters) and the DoV immunization targets to account for the performance of NIPs. Also, being a precursor descriptive study, NIP performance in countries affected by PHEs will be singularly analyzed without controlling for other third variables that may have influenced NIP performance. As such, the general scope of the study is limited in inferring a cause-effect relationship between PHEs and NIPs performance.

## Materials and Methods

### Study Design

A retrospective case study using secondary data on immunization indicators and PHEs.

### Study Population

Countries in the WHO Africa region that have received funding from the African Public Health Emergency Fund (APHEF) between 2012 and 2019 were used as case studies (28). It is important to note that the performance of NIPs in the selected countries is contingent on various factors, including the baseline start date for the different NIPs, the socio-economic contexts, national health budgets, political will, and leadership. Furthermore, the annual contributions made to the APHEF by WHO Africa countries and the basis for receiving funds during PHEs are determined by a defined set of country characteristics including income status, debt burden, equity, and level of poverty (28).

### Data Sources

**Figure 1:**
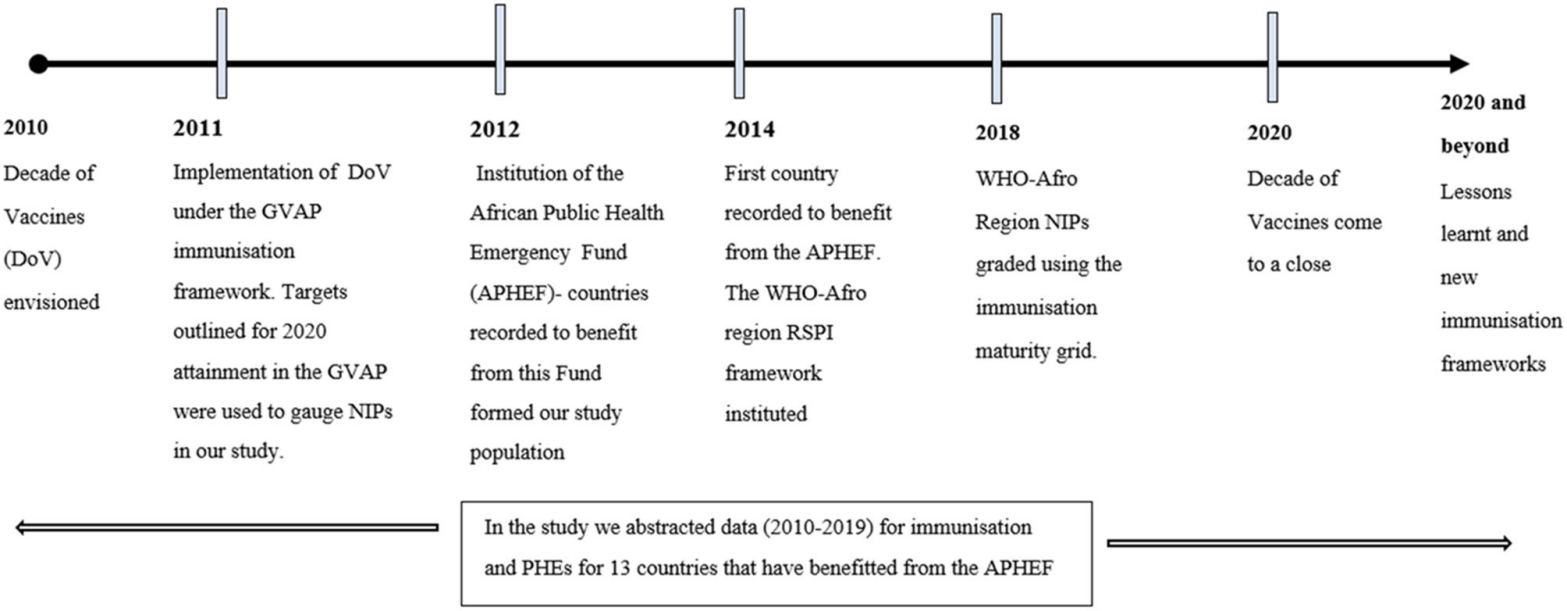
A conceptual framework of initiatives used to inform the choice of the study variables.

The study utilized retrospective secondary data from different sources available as of July 2020 and reported from 2010 to 2019 (Figure 1). The specified period is based on the DoV implementation timeline. Also, the APHEF, which was used to select the study population was instituted in 2012 (28) and is synchronous in timing with that of the DoV.

Data on immunization indicators outlined in the DoV were obtained through country reports made to the World Health Organisation (WHO) and the United Nations Children's Fund (UNICEF) using the Joint Reporting Form (JRF) (29).

The APHEF had supported three types of PHEs namely armed conflict, disasters, and disease outbreaks (28). Data was collated from different electronic databases reporting on these three types of PHEs. The databases used were:

a) The Emergency Events Database (EM-DAT) from the Centre for Research on the Epidemiology of Disasters that reports on natural and technological disasters, for the study these two disaster subtypes were collectively referred to as disasters (30).
b) The Uppsala Conflict Data Program (UCDP), a database that curates data on various types of armed conflicts namely state conflicts, one-sided violence conflicts, and non-state conflicts (31).
c) The WHO Emergency Preparedness and Response an integrated global alert and response system that reports on occurrences of disease outbreaks (32), which was corroborated with data from the Program for Monitoring Emerging Diseases Mail (PROMED- Mail) (33).

### The Determinant Variables

The counts of the three types of PHE namely disasters, armed conflicts, and disease outbreaks, and the total PHE count (the cumulative total count of the three PHE types) were used as the main determinant variables.

### Outcome Variables

The following selected immunization indicators implemented for the DoV (Figure 1) were used as the study outcome variables:

a) The combined Diphtheria, Tetanus, and Pertussis vaccine (DTP) coverage rates which were used as proxies to gauge the strength and reach of routine immunization programs (23) including:
  i) Having ≥90% national coverage for the third dose of the DTP (DTP3)
  ii) Drop-out rate between the first dose of the DTP (DTP1) and DTP3
b) Introduction of new and underutilized vaccines (23); three vaccines were considered for the study and used as proxies, they included two childhood vaccines- the Rotavirus vaccine and the Pneumococcal Conjugate Vaccine (PCV), and one adolescent vaccine-the Human Papillomavirus (HPV) vaccine.
c) The attainment status for the elimination of Maternal and Neonatal Tetanus (MNT) which is one of the targeted VPDs for regional and global elimination (23).
d) The establishment of national immunization technical advisory group (NITAG); which was used to gauge immunization prioritization and country ownership for NIPs. Data for this variable was obtained for both the existence and functionality of a NITAG. The functionality of a NITAG is pegged on an existing country NITAG meeting 6 minimum criteria as outlined by the WHO including the (A) administrative basis for the advisory group (B) formal written terms of reference (C) at least five different areas of expertise represented among NITAG core members (D) at least one meeting per year (E) circulation of the agenda documents at least 1 week before meetings, and (F) the mandatory disclosure of any conflict of interest (34). Each of the 6 outlined criteria needs to be satisfied for a country's NITAG to be merited as functional.
e) Immunization maturity grid which is a six-component tool used to rate immunization systems and identify gaps in the performance of NIPs. The six components used to rate the immunization maturity grid include (A) programme management and financing (B) immunization service delivery and new vaccine introduction (C) disease surveillance and VPD outbreak management (D) data management and analytics (E) vaccine quality, safety, and regulation and (F) community engagement (35).

### Data Synthesis and Analysis

To establish any underlying trends in the total PHE count for each of the study countries, the study duration was stratified into three-time points in 2010 at the beginning of the study, in 2014 after 5 years, and in 2019- 10 years and the end of the study.

The three types of PHEs were stratified by country using stacked bar plots in each of the study years to establish both the overall prevalence for each of the three types of PHEs and identify the individual country-specific prevalence for a given PHE.

All the statistical analysis and visualization of study data were done using the R software version 4.0.2 (R Core Team, 2020). The selected DoV immunization indicators were stratified by country and summarized using descriptive statistics such as the mean [Standard Deviation (SD)] and/or the median [Interquartile range (IQR)] for numerical continuous variables depending on their normality distribution, and counts and proportions, used for categorical variables. The results were presented in a table. We also used, a line graph to establish the temporal trends in the national and regional DTP3 coverage indicator.

Two groups were created for the target immunization indicators outlined in the DoV. The two groups were based on the number of years from 2010 to 2019 that each of the selected countries had either met a specific target or had failed to meet the target. Labels used for the two target groups were “target met”- Yes, and “target not met”- No. The distribution of the three types of PHEs and the total PHE counts were compared across the two groups using the Mann-Whitney U group-comparison test, as the data had a non-normal distribution. Statistical significance for the tests was defined at *p* < 0.05.

## Results

### Countries Benefiting From the APHEF

As of 2017, the APHEF had supported 13 countries within the WHO Africa region. The 13 countries were Angola, Burundi, Cameroon, the Central African Republic, the Democratic Republic of Congo, Ethiopia, Guinea, Liberia, Malawi, Niger, Sierra Leone, South Sudan, and Zimbabwe.

### PHEs Reported Between 2010 and 2019

**Figure 2:**
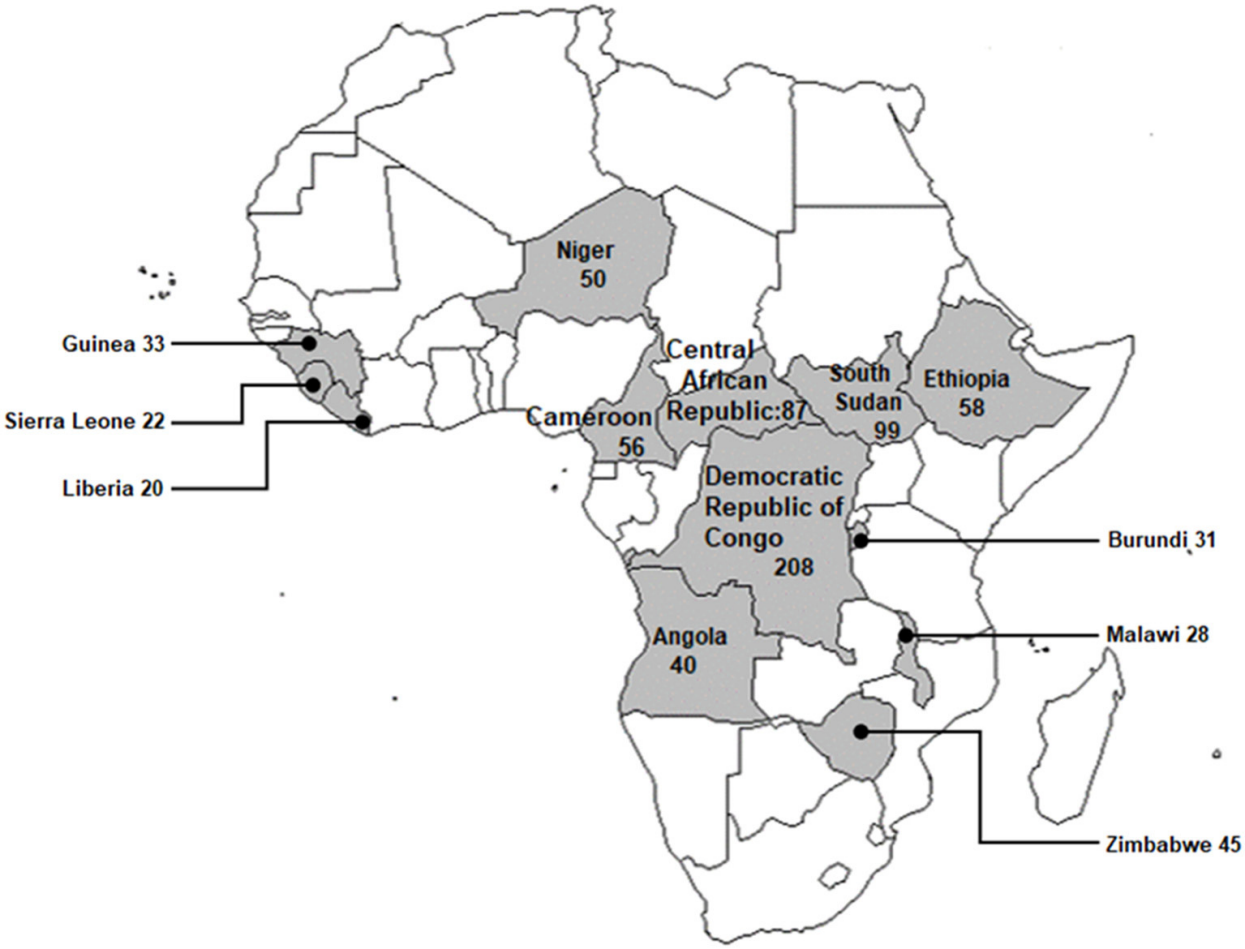
Distribution of total PHE counts by country from 2010 to 2019.

Between 2010 and 2019 PHEs were present in each of the study population countries and varied in count across the countries (Figure 2).

The highest total PHE count was recorded in the Democratic Republic of Congo (*n* = 208), while the lowest total PHE count was recorded in Liberia (*n* = 20) (Figure 2). Despite being the youngest country in the WHO Africa region, South Sudan (gained independence in 2011), had the second-highest total PHE count at (*n* = 99).

#### Total PHE Counts Across Three Time Periods

A snapshot of the total PHE count in the 13 countries in 2010, 2014, and 2019 showed the individual country trends at the beginning of the study period in 2010, after 5 years, and at 10 years in 2019. The PHE counts fluctuated across the study periods. While eight countries witnessed a decrease in the PHE count in 2014 compared to 2010, nine countries in 2019 recorded an increase in the PHE count compared to 2014 (Table 1).

**Table 1:** Total PHE counts stratified by year in 2010, 2014, and 2019.

**Country**	**2010**	**2014**	**2019**
Angola	5	1	4
Burundi	3	2	6
Cameroon	7	4	6
The Central African Republic	3	10	9
Democratic Republic of Congo	20	22	36
Ethiopia	5	2	11
Guinea	4	4	2
Liberia	2	1	1
Malawi	2	0	3
Niger	3	3	7
Sierra Leone	3	2	2
South Sudan^a^		8	13
Zimbabwe	6	5	2

*
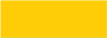
Increase in PHE count*.

*
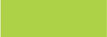
Decrease in PHE count*.

*
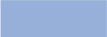
PHE count constant*.

*
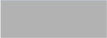
No data*.

a *South Sudan gained independence in 2011. As such data for 2010 is unavailable*.

#### Types of PHEs

**Figure 3:**
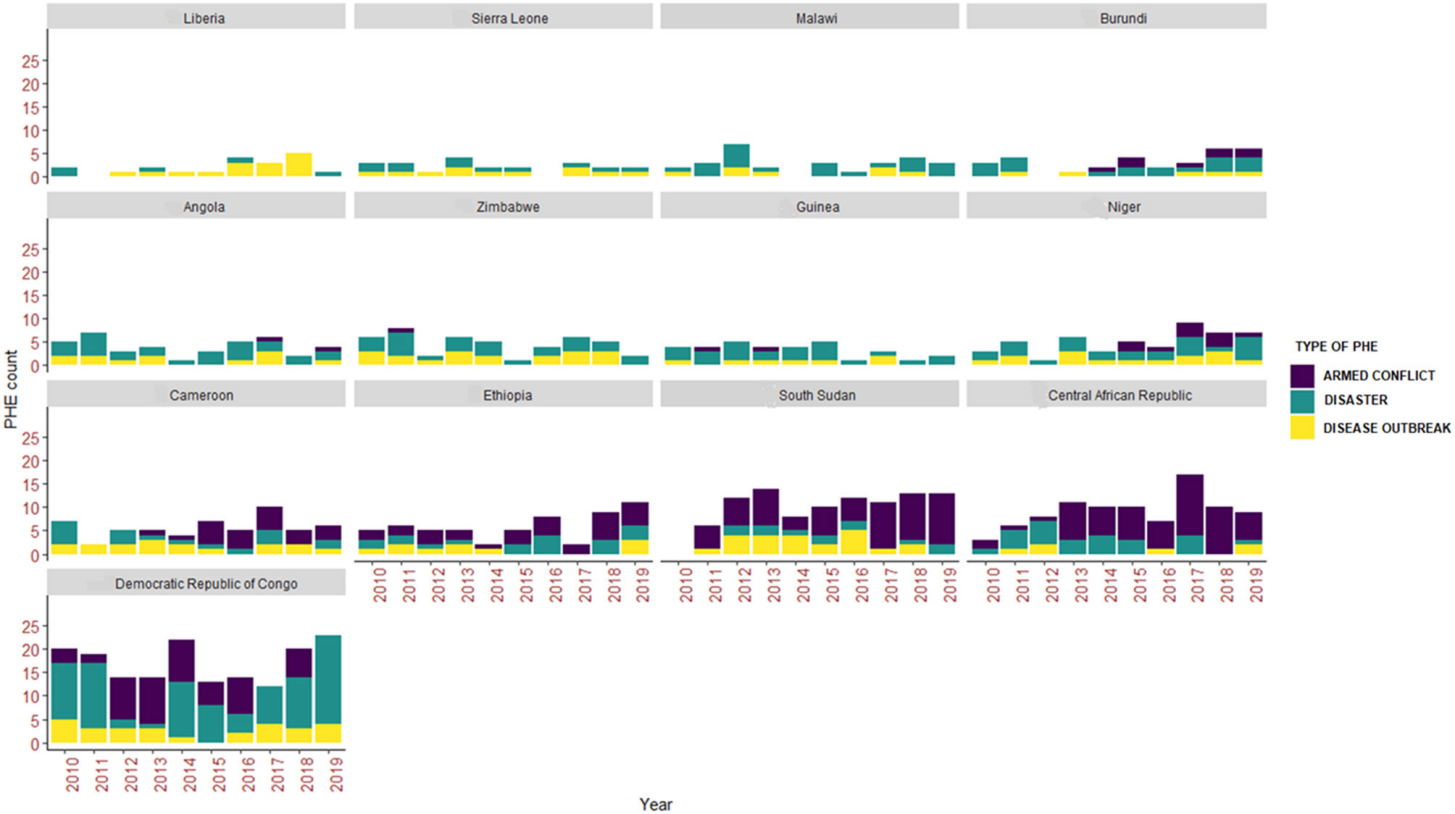
Three types of PHEs recorded in 13 WHO Africa region countries between 2010 and 2019.

Three types of PHEs namely armed conflicts, disasters, and disease outbreaks were extracted from databases and summarized (Figure 3). In the 13 countries and from 2010 to 2019, a total count of *n* = 175, *n* = 288, and *n* = 318 for disease outbreaks, armed conflicts, and disasters was recorded, respectively.

A summary of the annual distribution of the three types of PHEs occurring in each country during the study period is shown (Figure 3).

Overall, counts of armed conflicts (*n* = 89), disasters (*n* = 91), and disease outbreaks (*n* = 28) were highest in the Democratic Republic of Congo (Figure 3). In general, and with exception of the Democratic Republic of Congo, armed conflicts and disease outbreaks were majorly prevalent in all other countries for the entire study period (Figure 3).

### Performance of NIPs in the Period of PHEs

The target immunization indicators for the DoV were used to assess the performance of NIPs in the study countries from 2010 to 2019.

#### National DTP3 Coverage

The DTP3 coverage was used as a proxy indicator for the performance of NIPs in meeting national and regional immunization coverage targets.

**Figure 4:**
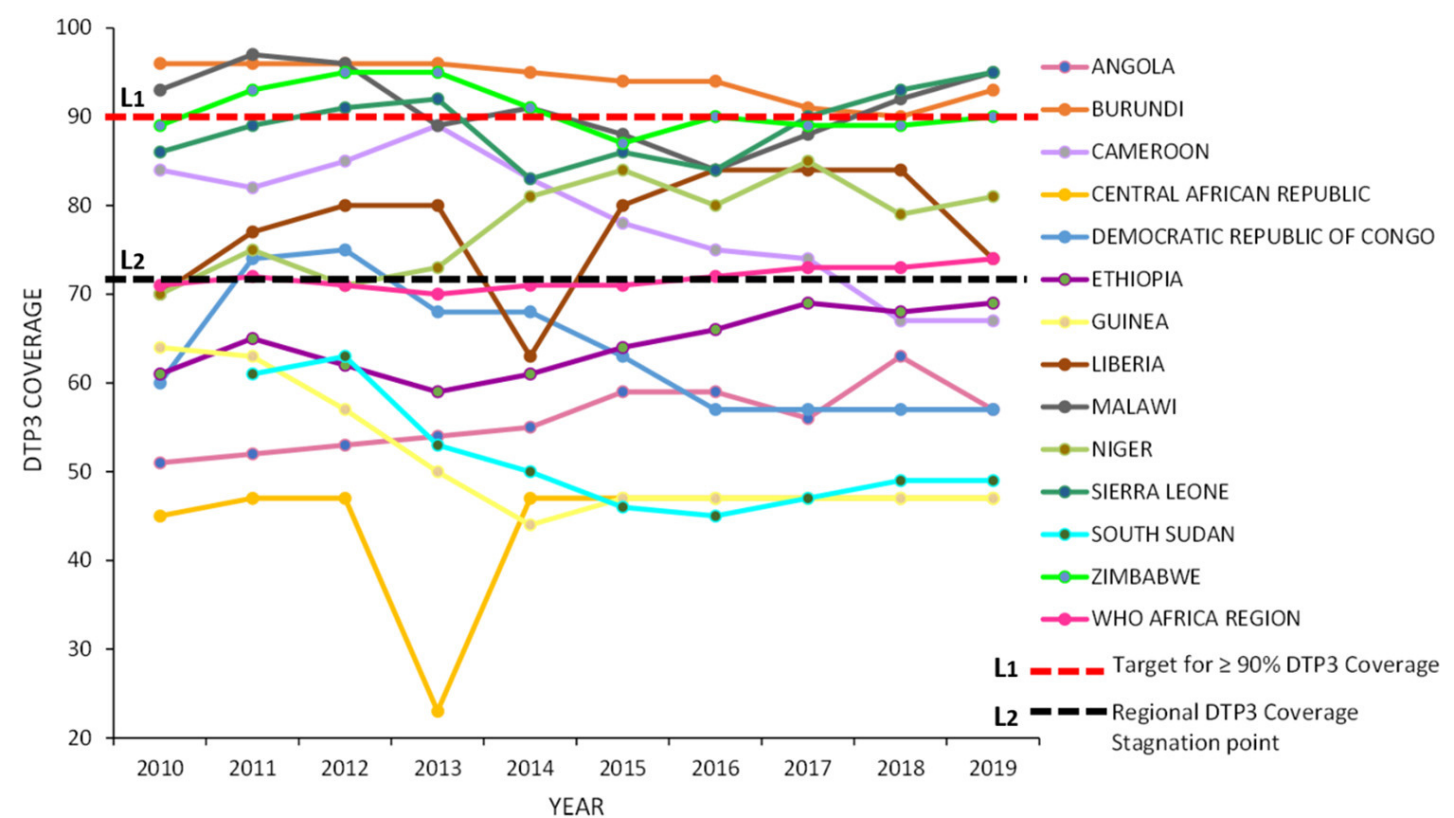
Annual DTP3 coverage trends between 2010 and 2019 for the 13 countries and the WHO Africa region.

The recorded trend in the annual national DTP3 coverage estimates in each of the 13 countries points to fluctuating patterns during the entire study period (Figure 4).

Only three (Burundi, Malawi, and Zimbabwe) of the 13 countries had a median national DTP3 coverage value that had met the ≥90% immunization coverage DoV target (Table 2). In addition to this, Burundi's annual DTP3 coverage rates were found to supersede the ≥90% immunization coverage target during the entire study period (Figure 4).

**Table 2:** Descriptive summary of NIP related immunization indicators between 2010 and 2019 stratified by country.

	**Angola**	**Burundi**	**Cameroon**	**CAR^a^**	**DRC^b^**	**Ethiopia**	**Guinea**	**Liberia**	**Malawi**	**Niger**	**Sierra Leone**	**South Sudan^c^**	**Zimbabwe**
	**Number of years data was obtained**												
**Immunization indicators**	***n =*** **10**	***n =*** **10**	***n =*** **10**	***n =*** **10**	***n =*** **10**	***n =*** **10**	***n =*** **10**	***n =*** **10**	***n =*** **10**	***n =*** **10**	***n =*** **10**	***n =*** **9**	***n =*** **10**
**DTP3 national coverage %:**													
Median	55.50	94.50	80.00	47.00	61.50	64.50	47.00	80.00	91.50	79.50	89.50	49.00	90.00
[IQR]	[5.25]	[2.75]	[9.50]	[0.00]	[11.00]	[6.25]	[8.25]	[8.25]	[6.25]	[7.50]	[5.75]	[6.00]	[3.50]
**Percentage drop-out rate between DTP1 and DTP3:**													
Median	15.00	3.00	8.00	24.00	6.00	5.00	6.00	8.50	4.50	7.50	5.50	17.00	4.50
[IQR]	[1.75]	[0.75]	[0.75]	[9.50]	[3.50]	[2.50]	[9.00]	[4.50]	[2.50]	[5.50]	[3.25]	[14.00]	[1.75]
Total number of new and underutilized vaccines introduced as of 2019 (Rotavirus, PCV and HPV vaccines)	2	2	2	1	2	3	0	3	3	2	2	0	3
Number of years a country has had an existing NITAG:													
Years (%)	6 (60)	1(10)	5 (50)	1(10)	4 (40)	7 (70)	2 (20)	0 (0)	5 (50)	8 (80)	4 (40)	7 (77.80)	10 (100)
Number of years a country has had a functional NITAG:													
Years (%)	0 (0)	0 (0)	2 (20)	0 (0)	2 (20)	4 (40)	1 (10)	0 (0)	3 (30)	2 (20)	3 (30)	3 (33.30)	4 (40)
Has country eliminated MNT	No	Yes	Yes	No	Yes	Yes	No	Yes	Yes	No	Yes	No	Yes
Number of years a country has been MNT free within the study period	0	10	8	0	2	2	0	9	10	0	7	0	10

a *CAR Central African Republic*.

b *DRC Democratic Republic of Congo*.

c *South Sudan gained independence in 2011 hence data obtained were for 9 years*.

The WHO Africa region DTP3 coverage between 2010 and 2019 stagnated at around the average 72% mark (Figure 4). Six countries, (the Central African Republic, the Democratic Republic of Congo, South Sudan, Guinea, Angola, and Ethiopia) had national DTP3 coverage rates below the regional DTP3 stagnation point in the entire study period (Figure 4). While four countries maintained a coverage rate above that of the region including Burundi, Malawi, Zimbabwe, and Sierra Leone. The remaining three countries (Liberia, Cameroon, and Niger) had fluctuating immunization coverage rates.

Two countries were recorded to have major dips, followed by major increases in their national DTP3 vaccine coverage in some of the study years; they were the Central African Republic and Liberia, these dips occurred in 2013 and 2014, respectively (Figure 4).

#### Percentage Drop-Out Rates Between DTP1 and DTP3

To gauge accessibility and utilization of immunization services we used drop-out rates between DTP1 and DTP3. Three countries (The Central African Republic, South Sudan, and Angola) had drop-out rates above 10%. The highest drop-out rate in the 10-year study period was recorded in the Central African Republic, with a median [IQR] of 24.00 [9.50] (Table 2).

#### Introduction of New and Underutilized Vaccines

To gauge the progress of countries in the introduction of new vaccines in their NIPs three vaccines: The Rotavirus, PCV, and the HPV vaccines were considered. The PCV and the Rotavirus vaccine were first introduced in the WHO Africa region between the years 2008 and 2009, while the HPV vaccine was first introduced in 2014. Among the thirteen countries, eleven countries had introduced PCV, while seven countries had introduced the Rotavirus vaccine to their immunization schedule between 2010 and 2014. Only four countries had introduced the HPV vaccine in the second half of the study period (2015–2019). The HPV vaccine is the newest to be introduced in the NIPs of countries in the WHO Africa region.

Ethiopia, Liberia, Malawi, and Zimbabwe had introduced all three vaccines by 2019. While Guinea and South Sudan were yet to introduce any of the three vaccines in their NIPs by 2019 (Table 2).

#### Elimination of MNT

To monitor the performance of countries in meeting global and regional elimination targets for key VPDs the elimination of MNT was considered. As of 2019, 8 of the 13 countries had attained MNT elimination. Countries yet to eliminate MNT by 2019 were Angola, the Central African Republic, Guinea, Niger, and South Sudan (Table 2).

#### Establishment of NITAGs

To gauge progress toward country ownership of NIPs and immunization prioritization, the establishment of NITAGs in the study countries was examined during the entire study period.

Except for Zimbabwe, none of the countries had an existing NITAG for the entire 10-year study period. Niger, South Sudan, Ethiopia, and Angola had existing NITAGs for over half of the study period. The remaining countries had existing NITAGs for less than half of the study period, apart from Liberia which had no existing NITAG for the 10-year study period (Table 2).

All 13 study countries had functional NITAGs for less than half of the study period (Table 2).

#### Immunization Maturity Grid

The immunization maturity grid is a six-component tool developed by the WHO aimed at attaining control, elimination, and eradication of key VPDs in the African continent. As of 2018, the NIPs of the 13 countries were classified into four maturity levels as shown in Table 3.

**Table 3:** Classification of countries according to their immunization maturity grid.

**Immunization maturity grid**	**Countries in the maturity grid**	**Rating criteria^a^**
Level 1	The Central African Republic, South Sudan, Sierra Leone, and Liberia	Countries with very weak immunization systems
Level 2	Guinea, Angola, and the Democratic Republic of Congo	Countries with significant deficiencies in immunization service delivery
Level 3	Cameroon, Niger, Ethiopia, and Malawi	Countries with targeted areas for improvement in their immunization programs
Level 4	Burundi and Zimbabwe	Countries with strong and robust immunization systems

a *Adopted from the Business case for WHO immunization activities on the African continent 2018 (35)*.

### The Association Between PHEs and NIP Performance

We compared the distribution in count for the three types of PHEs and the total PHEs across the two groups created from the DoV immunization targets.

In the 13 countries from 2010 to 2019, higher armed conflict counts were associated with not meeting the immunization targets for national DTP3 coverage of ≥90% and MNT elimination, *p* < 0.01 (Table 4).

**Table 4:** A Comparison of PHE count across two target groups of immunization indicators outlined in the DoV.

	**Target for ≥90% national DTP3 coverage**			**Target for MNT elimination**			**Target to have a functional NITAG**			**Target to introduce at least one new or underutilized vaccine^b^**		
	**Target not met**	**Target met**	***p-*** ***value***	**Target not met**	**Target met**	***p-*** ***value***	**Target not met**	**Target met**	***p-*** ***value***	**Target not met**	**Target met**	***p-*** ***value***
	***n*** **^a^ =102**	***n*** **^a^ =27**		***n*** **^a^ = 71**	***n*** **^a^ = 58**		***n*** **^a^ = 105**	***n*** **^a^ = 24**		***n*** **^a^ = 103**	***n*** **^a^ = 24**	
**PHE type**												
**Armed conflict**												
Median	1	0	<0.01^*^	1	0	<0.01^*^	0	0.5	0.40	1	0	0.05
[IQR]	[5]	[0]		[5.5]	[1]		[3]	[5]		[3]	[1]	
Disaster												
Median	2	2	0.90	2	1	0.03^*^	2	2	0.30	2	2	0.60
[IQR]	[2]	[2]		[2.5]	[2]		[2]	[1]		[2]	[2]	
**Disease outbreak**												
Median	1	1	0.10	1	1	0.90	1	2	0.40	1	1	0.60
[IQR]	[2]	[1.5]		[2]	[2]		[2]	[3]		[2]	[1]	
**Total PHEs^c^**												
Median	5	3	<0.01^*^	5	3	<0.01^*^	4	5	0.10	5	5	0.06
[IQR]	[6]	[2]		[7]	[3]		[5]	[6.5]		[3]	[3]	

* *p-value significant*.

a *Between 2010 and 2019 each of the 13 countries contributed 10 years, except for South Sudan (gained independence in 2011), which contributed 9 years, hence, the total number of years for all the 13 countries was 129*.

b *For this target 2 years had no data as two countries had already introduced all the three new vaccines examined in the study in their preceding years*.

c *This variable is a function of the total count of the armed conflict, disaster, and disease outbreak variable*.

Higher disaster counts were also, associated with not meeting the target for MNT elimination, *p* = 0.03 (Table 4).

Higher total PHE counts were associated with not meeting immunization targets for national DTP3 coverage of ≥90% and not attaining MNT elimination, *p* < 0.01 (Table 4).

Higher counts of armed conflicts, disasters, disease outbreaks, and total PHEs were not associated with meeting the DoV immunization targets for introducing new vaccines and the presence of functional NITAGs, *p* > 0.05 (Table 4).

## Discussion

The primary intent of the study was to characterize the NIPs of countries within the WHO Africa region experiencing PHEs using immunization targets outlined for the DoV as proxy indicators. We found that PHEs were endemic during the entire study period and the performance of NIPs in the PHE-endemic countries was variable, although mostly suboptimal. Additionally, higher total PHE and armed conflict counts were associated with not achieving the immunization targets for ≥90% national DTP3 coverage and MNT elimination. Higher disaster counts were also associated with not eliminating MNT.

The endemicity of PHEs observed during the study period, agrees with other reports on the recurrent and diverse nature of PHEs experienced in the WHO Africa region (3, 36). While armed conflict and disasters were found to have the highest cumulative count in our study, contrary, trends were observed in the WHO Africa region, with a majority of reported PHEs being that of disease outbreaks (3, 36, 37). The discrepancy can be explained by findings from other studies done in similar settings, where disease outbreaks were noted to occasionally go unrecorded as they occur as a twin PHE, in the context of armed conflicts and disasters (2, 38).

Regardless of the immunization maturity level, the overall performance of NIPs in the PHE endemic countries was variable but predominantly suboptimal. The fluctuating and the low national DTP3 coverage rates below the WHO Africa region DTP3 coverage stagnation point are synonymous with DTP3 coverage reports in other NIPs in the region during PHEs (11, 21). As outlined by Grundy et al. in their study, other WHO Africa region countries like Nigeria and Somalia that have experienced constant civil unrest and were yet to benefit from the APHEF were reported to similarly have sub-optimal DTP3 coverage rates (6). Similarly, the high DTP vaccine drop-out; classified as ≥10% (24), recorded in some of the NIPs, concur with findings from a study by Mugali et al. (39) in Afghanistan where high DTP drop-out rates were associated with conflict and insecurity.

The introduction of the PCV and the Rotavirus vaccines into the NIPs of the study countries was high compared to the HPV vaccine. These findings are comparable to those in the region as 86, 76, and 8% of GAVI eligible countries had introduced the PCV, Rotavirus, and the HPV vaccines, respectively, by 2017 (40). Delay in HPV vaccine introduction has been attributed elsewhere to the poor adoption of life-course immunization, vaccine supply constraints, and pricing issues (16, 22) which stand exacerbated by PHEs (6). The outlier countries yet to introduce any of the three selected vaccines are potential key pointers to the contributing role PHEs play in delaying vaccine introductions into NIPs as raised by Grundy et al. in their study (6).

Despite the positive progress in NITAG establishment in the WHO Africa region (41), a non-consistent pattern in their existence and functionality simultaneously existed. Such inconsistent trends have been attributed in previous reports to the lack of political commitment and country ownerships of NIPs and the low NITAG financial investments, in general, and during PHEs (34, 41).

The elimination target for MNT was not met in countries with high counts of PHEs like South Sudan, the Central African Republic, Niger, Guinea, and Angola. The enlisted five countries constitute nearly 50% of the remaining global MNT elimination priority countries (42) and have similar trends with other MNT priority countries like Afghanistan and Yemen, where the presence of protracted PHEs has been blamed on delaying elimination (43).

Having higher armed conflict and total PHE counts were associated with not attaining the target for ≥90% national DTP3 coverage. These findings are comparable to those from studies conducted in similar contexts where armed conflicts were reported to be associated with poor immunization coverage outcomes (6, 11, 27). Conversely, while the Democratic Republic of Congo had the highest PHE count, its national DTP3 coverage was not the poorest. As described elsewhere, this finding in the Democratic Republic of Congo could be partly attributed to the humanitarian aid in the country (44). In addition, there have been recent specific plans developed by the government of the Democratic Republic of Congo in collaboration with technical and financial partners such as WHO and GAVI, The Vaccine Alliance to strengthen the NIPs in the country (45).

The findings further show that periods of higher counts of armed conflicts, disasters, and total PHEs were also associated with not eliminating MNT. Elimination of MNT in the WHO Africa region has been evasive to some extent, with most of the countries prioritized for MNT elimination being affected by PHEs (46, 47). In other PHE endemic contexts, where MNT elimination remains unattained, low tetanus vaccine coverage among women of reproductive age, unhygienic birth practices, delayed treatment, and inadequate MNT surveillance have been cited to delay progress (46, 48).

Higher counts of all the three types of PHEs and the total PHEs were not associated with meeting the immunization targets of having functional NITAGs or the introduction of new vaccines. The lack of consistency in the existence and functionality of NITAGs is not uncommon as observed in other PHE-prone contexts in the region (41). On the other hand, during PHEs, whereas the functionality of NITAGs may be compromised, the WHO recommends humanitarian immunization teams to utilize the unique expertise and local knowledge of existing NITAG members (17). For the target to introduce new vaccines, a possible caveat may exist in interpreting association, as within the scope of this study, only three vaccines were selected out of a pool of new vaccines. Conversely, we equally recognize that despite PHE endemicity, most of the case study countries had performed relatively well in this target which is also credited as one of the WHO Africa region DoV successes owing to political commitment and strong immunization investments (22). Arguably, such progressive performance noted in this DoV target, can offer a lesson; that the link between PHEs and immunization performance may not be always absolute.

In summation, it is imperative to note that central to the attainment of the discussed DoV targets and “health for all” as embodied under Sustainable Development Goal 3 (SDG 3) is the presence of peace and stability in a country which is a critical pillar to SDG 16 namely “peace justice and strong institutions” (49, 50). This will reciprocally ensure that every individual regardless of their background will have access to vaccines (50).

Our results should be interpreted in light of certain caveats. First, our study was limited to three broad types of PHEs, that may not be exhaustively representative of all types of PHE experienced in the study countries. Furthermore, it would have been useful to provide information on the duration of each of the PHEs assessed per country. However, owing to the retrospective and longitudinal design of this study, as well as the incomplete and inconsistent reporting of PHE data, the severity, and the duration of the PHEs, could not be analyzed within the scope of this study. It should be noted that PHEs often overlap in the real-world context, and it may be challenging to identify how a single PHE may have impacted the performance of immunization programs. Additionally, we may be unable to determine how previous PHEs experienced before the start of the study period in 2010 may have affected the NIPs in the selected countries.

Secondly, we used country-generated reports from the WHO/UNICEF JRF to abstract data on DoV immunization indicators. It is, possible that potential inaccuracies in the country data may have influenced the outcome of our study. However, this could not be avoided within the scope of this study. Data quality issues from country reports have been constantly highlighted as one of the major concerns by the WHO, SAGE in their annual DoV immunization reports (22, 26). Similarly, since our study used immunization indicators outlined for the DOV between 2010 and 2020 to assess NIP performance, analyzing pre-PHE years immunization data was beyond the scope of our study owing to differences in immunization goals between the pre-PHE period and the PHE period. Additionally, data on NIP immunization maturity was first availed in 2018 in the WHO Africa region (35), as such the study is unable to account for NIP maturity data for the period preceding 2018.

Lastly, while associations between the occurrence of PHEs and the performance of immunization programs were highlighted we cannot rule out with certainty the influence of other third variables on immunization program performance. It is also, worth noting several confounders may have affected the performance of NIPs in addition to PHEs which we were unable to account for, owing to the limited scope of the study in inferring a cause-effect relationship between PHEs and NIP performance. Similarly, it was not possible to account for emergency responses aimed at providing immunization services during PHEs. As such, our estimated results may represent a lower bound for the true effect of PHEs on immunization.

Future research investigations should address these limitations to broaden the scope of evidence on the interactions that exist between PHEs and immunization performance. We propose that future studies should further explore (1) how other types and grades of PHEs and the duration of PHEs may variably impact immunization performance, (2) immunization performance at the sub-national level using immunization data from localized communities where PHEs are recorded to occur, (3) how other third variables including NIP performance confounders may act together with PHEs to influence immunization performance, and (4) the analysis of PHEs and immunization performance in other WHO regions in comparison with the findings from the WHO Africa region.

## Conclusion

PHEs are endemically present in the WHO Africa region and form part of the eco-system in which NIPs exist. With the goal of extending immunization to all individuals, countries experiencing PHEs within the WHO Africa region may be excluded from reaping the full benefits of immunization owing to unmet targets. Immunization performance in countries where PHEs are endemic are largely suboptimal and not at par with envisioned immunization targets for the DoV. As we transition to the Immunization Agenda 2030, PHEs like the COVID-19 pandemic, armed conflicts, and disasters are a major threat to NIPs. Therefore, priority should be given to developing evidence-based interventions to mitigate the impacts of PHEs on NIPs. Such interventions may include the commitment by governments in the WHO Africa region to strengthen the resilience of NIPs against PHE challenges by investing in PHE prevention and mitigation initiatives like the APHEF. Characterizing the performance of NIPs in PHE contexts is undoubtedly elemental in bridging the gap to equitable access to immunization for all populations, irrespective of where they live, thus enabling the collective achievement in shared goals like the DoV, SDG 3 and 16, and the Immunization Agenda 2030, thereby, enhancing regional health outcomes.

## Data Availability Statement

The original contributions presented in the study are included in the article/supplementary material, further inquiries can be directed to the corresponding authors.

## Author Contributions

BK conceived of the presented idea, provided critical feedback, and helped shape the manuscript. VC extracted and analyzed the data and wrote the manuscript. EA-D and ECH contributed to the development of the methods and coherence of the manuscript. All authors read, edited, and approved the final manuscript.

## Conflict of Interest

The authors declare that the research was conducted in the absence of any commercial or financial relationships that could be construed as a potential conflict of interest.

## Publisher's Note

All claims expressed in this article are solely those of the authors and do not necessarily represent those of their affiliated organizations, or those of the publisher, the editors and the reviewers. Any product that may be evaluated in this article, or claim that may be made by its manufacturer, is not guaranteed or endorsed by the publisher.

## Footnotes

^1^Disaster- Is described as an event that results in serious disruption of the functioning of a community or a society causing widespread human, material, economic or environmental losses which exceed the ability of the affected community or society to cope using its own resources. Available from: https://www.emdat.be/Glossary.
